# Beyond Passive Immunity: Is There Priming of the Fetal Immune System Following Vaccination in Pregnancy and What Are the Potential Clinical Implications?

**DOI:** 10.3389/fimmu.2018.01548

**Published:** 2018-07-16

**Authors:** Christopher R. Wilcox, Christine E. Jones

**Affiliations:** ^1^NIHR Clinical Research Facility, Southampton Centre for Biomedical Research, University Hospital Southampton NHS Foundation Trust, Southampton, United Kingdom; ^2^Faculty of Medicine, Institute for Life Sciences, University of Southampton, University Hospital Southampton NHS Foundation Trust, Southampton, United Kingdom

**Keywords:** vaccination, pregnancy, fetus, priming, antigen, *in utero*, immunity

## Abstract

Infection is responsible for over half a million neonatal deaths worldwide every year, and vaccination in pregnancy is becoming increasingly recognized as an important strategy for the protection of young infants. Increasing evidence suggests that exposure to maternal infection *in utero* may “prime” the developing immune system, even in the absence of infant infection. It is also possible that *in utero* priming may occur following maternal vaccination, with antigen-specific cellular immune responses detectable *in utero* and at birth. However, this remains a topic of some controversy. This review focuses on the evidence for *in utero* priming and the clinical implications for vaccination in pregnancy, considering whether *in utero* priming following vaccination could provide protection independent of antibody-mediated passive immunity, the possible effects of vaccination on subsequent infant vaccinations, their potential “non-specific” effects, and how the design and timing of vaccination might affect prenatal priming. Looking forward, we describe other possible options for quantifying antigen-specific cellular responses, including MHC tetramers, novel proliferation and cytokine-based assays, and animal models. Together, these may help us address future research questions and establish more robust evidence of fetal immune system priming.

## Introduction

Neonates have an inexperienced immune system and infection is responsible for over half a million neonatal deaths worldwide every year (1). Our current understanding of the functional mechanisms underlying the perinatal and neonatal immune systems remains incomplete (2). Improving this understanding is crucial for improving infant survival rates, and for the optimization of interventions, including vaccination in pregnancy and in early life. Vaccination of neonates is challenging as they may mount inadequate protective immunity, and the presence of maternal antibodies may blunt vaccine responses (3, 4). Vaccination in pregnancy works by boosting the concentration of maternal vaccine-specific antibody, and thus the quantity transported to the fetus across the placenta (5). This can provide effective protection for the newborn until the period of greatest vulnerability has passed, or until the time of routine infant vaccinations.

Evidence suggests that priming of the fetal immune system may occur in response to maternal infections, environmental and food allergens, and maternal vaccination, with studies showing evidence of adaptive antigen-specific cellular immune responses *in utero* and at birth. However, this remains a topic of some controversy, and our understanding of the underlying mechanisms and clinical implications for vaccination in pregnancy and subsequent infant vaccinations remains poor. In this review, we aim to summarize our current understanding of this field and highlight areas where further research would be most beneficial.

## Transfer of Infectious Antigens and Allergens During Pregnancy

It is now well established that maternal infection during pregnancy can affect the fetal immune system, even in the absence of vertical transmission of pathogens. Maternal infection may alter the susceptibility of infants to later childhood diseases, their response to vaccination, and the development of immunopathological disorders (6, 7). Furthermore, there is growing evidence that such exposure *in utero* may “prime” the developing immune system, even in the absence of infant infection, resulting in a more activated and mature immunophenotype (8).

One of the first studies to suggest this phenomenon, published in 1972, followed 12 Eskimo children 10 years after intrauterine exposure to mumps virus during an epidemic (9). None of the children had evidence of mumps neutralizing antibodies, yet 10 had positive skin tests, which the authors suggested was evidence of fetal cellular immune sensitization which persisted into childhood. Since this time, *in utero* priming has been suggested by both animal models (10) and in studies of uninfected children born to mothers with a range of infections. Many of these studies have been conducted in infants who were HIV exposed, but remained uninfected. Compared with unexposed infants, a proportion of these infants show enhanced immune activation with a lower percentage of naïve T cells and higher proportion of central memory T cells demonstrating markers of differentiation and senescence, as well as HIV-specific immune responses at birth (11–15).

Other examples suggesting that priming may occur as a result of maternal infection include studies of cytomegalovirus (16), *Mycobacterium tuberculosis* (8), hepatitis B (17), hepatitis C (18), and *Plasmodium falciparum* (19). In endemic regions, *in utero* sensitization to helminths has also been demonstrated by the detection of fetal lymphocyte responses to parasite antigens and the detection of specific immunoglobulins in cord blood. These include filariasis (20), schistosomiasis (21), onchocerciasis (22), and ascariasis (23). *In utero* exposure to helminths may also influence the neonatal response to subsequent vaccinations. An early study by Malhotra et al. compared infants sensitized, or not sensitized, to helminth antigens *in utero*, and demonstrated that helminth-specific immune responses persisted into childhood. Furthermore, prenatal sensitization biased T cell immunity induced by BCG vaccination away from type 1 IFN-γ responses, which are associated with protection against mycobacterial infection (24). However, the underlying mechanisms are complex, and more recent studies investigating whether or not there is a significant suppressive effect of prenatal exposure to maternal parasitic infections on infant vaccine responses (including *Haemophilus influenzae* type B, diphtheria, and BCG vaccines) have shown conflicting results (25, 26).

It should be noted that our understanding of how the fetal immune system actually gets primed by maternal antigen in the absence of fetal infection remains unclear. Low levels of vertical transmission of antigen are possible, and it may be that maternal cells or antigen-loaded microvesicles transverse the placental barrier, followed by later clearing (8, 13). It has also been suggested that transplacental transport of pathogen-derived antigen may occur in the form of immune complexes, mediated by the neonatal Fc receptor (FcRn) (27–29). Early studies examining tetanus toxoid using a dual *ex vivo* placental perfusion model identified tetanus antigen in both the maternal and fetal circulations (27, 28). They noted that the ratio of antigen to antibody in the maternal circulation closely matched that observed in the fetal circulation, suggesting a coupling of antigen transfer to the transport of antibody. More recently, May et al. (29) studied the transplacental transfer of *P. falciparum* merozoite surface protein 1 (MSP1), the most abundant malaria blood stage antigen (30). MSP1 was frequently found in the cord blood of offspring to malaria-infected women and was often complexed to antibody. Furthermore, using the placental perfusion model, they demonstrated that immunoglobulin G (IgG)-bound MSP1 was present in the fetal perfusate, and confocal laser scanning microscopy revealed MSP1 in the fetal villous stroma, predominately the fetal endothelial cells. How such immune complexes can pass through the fetal endothelial cells into the fetal circulation, however, remains unknown. Finally, another possibility is that the fetal immune system may not necessarily be directly affected by contact with infectious antigens, but from exposure to a maternal immune system under the influence of infection (8, 13). Transplacental transfer of maternal inflammatory mediators, such as cytokines and chemokines, could lead to fetal T cell activation and differentiation. However, this issue is contentious, as recent placental perfusion studies directly investigating whether or not there is any vertical transfer of cytokines have shown conflicting results (31, 32).

The mechanisms underlying the fetal immune response to such exposure *in utero* are also poorly understood. It is proposed that exposure stimulates fetal innate immune cells, including dendritic cells and macrophages, to produce acute phase cytokines (such as IL-1β), and directs the innate and adaptive immune systems toward an inflammatory response and promotion of fetal T cell priming (14). How the fetal immune system might respond differently to maternal antigen and/or cytokine exposure remains to be seen. With regards to the clinical consequences of such exposure, it is possible that infants may acquire protective immune responses, but alternatively they may also develop immune tolerance, increasing their susceptibility to both homologous and unrelated pathogens (6, 29). Possible mechanisms of immune tolerance include T cell anergy (33) and the development of expanded populations of regulatory T cells, which have been shown to suppress antigen-specific immune responses to malaria in infants born to mothers with infection during pregnancy (34, 35). Improving our understanding of this area may therefore have important implications for the screening and management of maternal infections during pregnancy.

Whether *in utero* priming occurs in response to environmental allergens (including food and airborne allergens, such as the house dust mite) and whether this contributes toward the development of atopy in the neonate, remains a subject of significant controversy. There is likely to be at least some degree of fetal allergen exposure (36) and allergen-specific IgE is indeed detectable in cord blood. However, it remains debatable whether this is of fetal or maternal origin (37–39), and there is recent evidence to suggest that this may be predominantly maternal IgE transported across the placenta as IgG/IgE complexes (40). Furthermore, among studies focusing on the development of allergen-specific T-helper populations, it remains unclear whether observed cord blood mononuclear cell responses to such exposure necessarily reflect *in utero* sensitization (41–44). These findings may have important clinical implications for the etiology for the atopic disease, and for the development of primary prevention strategies (45) including maternal allergen avoidance during pregnancy, for which there is limited evidence of protective benefit to date (46).

## Transfer of Vaccine-Specific Antigens During Pregnancy

Transplacental transfer of IgG during pregnancy provides passive immunity for the newborn and is crucial for protection against infection in early life. Transcytosis of IgG occurs *via* pH-dependent binding with FcRn at the placental syncytiotrophoblast layer (47). IgG is taken up by endocytosis and then binds with FcRn within the acidic environment of early endosomes, where it is protected from proteolytic degradation (48, 49). IgG is then transcytosed to the basal surface and becomes dissociated from FcRn upon a return to physiological pH. Vaccination in pregnancy works by boosting the concentration of maternal vaccine-specific antibody, and thus the quantity transferred across the placenta to the infant. A number of recent trials have demonstrated that this strategy is safe and efficient means of protecting mother, fetus, and infant from infection (50–53) and several countries now routinely offer vaccination to pregnant women against influenza, pertussis, and tetanus (54).

There is some evidence that the fetal immune system may be influenced as a result of vaccination by more than just the passive immunity provided through IgG transfer. As with infectious disease antigens, it may also be sensitized *in utero* to vaccine antigens to which the mother has been exposed during pregnancy; however, research in this area remains somewhat scarce. Historically, B cell responses have been studied indirectly by comparing levels of anti-vaccine IgM and IgG antibodies present in cord blood between mothers who were vaccinated and non-vaccinated. Given that IgM does not cross the placental barrier, any differences might suggest that there was sensitization of fetal B lymphocytes. Early studies in the 1980s of tetanus vaccination during pregnancy reported the identification of toxoid-specific IgM in some infants (55) and later work established that the detection of IgM in cord blood was most common when women had undergone vaccination in the second or third trimester (56). Vaccine-specific IgM in cord blood has also been identified following influenza vaccination (57, 58); however, to the best of our knowledge, there is no published data in this area for pertussis vaccination.

More recently, direct measurement of vaccine-influenced fetal T cell priming has been achieved by Rastogi et al. using MHC tetramers to compare the cord blood of infants born to influenza vaccinated and non-vaccinated mothers (58). MHC class I and II tetramers permit the detection of antigen-specific T cells at the single-cell level using flow cytometry (59). They contain four linked human leukocyte antigen (HLA) molecules loaded with a peptide, and this MHC–peptide complex is recognized by a specific subset of T cells *via* the T cell receptor (60). MHC tetramers are able to differentiate between naive and memory T cells on the basis of their expression of either the low- or high-molecular weight isoforms of the leukocyte common antigen, CD45RO or CD45RA, respectively (61). Cord blood T cells are usually considered to be predominantly naïve, due to their low expression of CD45RO (62, 63).

In their study, Rastogi et al. demonstrated that the influenza-specific cord T cells in this study were repeatedly CD45RO+, suggesting an effector memory T cell response. Some studies have argued that the antigen-specific fetal T cells observed are not necessarily conventionally primed T-helper memory cells, but instead might represent a transitional population between thymocytes and adult T cells known as “recent thymic migrants” (64). These cells dominate the human peripheral T cell compartment during the neonatal period and are able to quickly generate T-cell cytokine signals in the absence of conventional T cells (65). However, the T cell phenotype observed in the study by Rastogi et al. is not consistent with recent thymic migrants, and instead suggests successful generation of an effector memory T cell response as a result of *in utero* priming by maternal influenza vaccination.

## What is the Potential Clinical Relevance of These Findings for Vaccination in Pregnancy?

It is possible that *in utero* priming following vaccination could benefit the neonate by providing protection independent of antibody-mediated passive immunity. This may be particularly important for infections mediated by memory T cell immunopathology such as respiratory syncytial virus (RSV) (66–68). RSV is the leading viral cause of lower respiratory tract infection in infants and a major cause of childhood morbidity and mortality globally (69). While no vaccine against RSV is yet approved for use in pregnancy, a number of candidates are currently in development, one of which is undergoing international phase III efficacy trials in pregnant women (NCT02624947) (54). For such an infection, providing passive immunity alone through generation of high-antibody titers will likely be insufficient to prevent disease in every individual (70). Yet, results from both mouse models and experimental human challenge studies suggest if vaccines elicit RSV-specific memory CD8 T cell responses; this may promote more effective viral clearance upon infection and promote longer-lived immunity (66, 71).

It has also been established that presence of maternal vaccine-induced antibodies can interfere with the concentration of subsequent infant vaccine responses (3, 4). Vaccination in pregnancy against pertussis, for example, reduces infant morbidity and mortality from whooping cough (51) but also reduces the antibody response to infant pertussis vaccination (72, 73). The effect of vaccine-induced *in utero* fetal T cell priming on subsequent infant T cell responses to postnatal vaccines is unknown; however, to the best of our knowledge, this blunting effect has not been described for infants’ cellular immune responses to date (74). Furthermore, the clinical significance of blunting of infant antibody responses is still poorly understood.

It is also becomingly increasingly recognized that vaccination may have immune modulatory effects beyond initiating antigen-specific adaptive responses, termed “non-specific effects” (75). Recent randomized and observation studies in Africa have shown non-specific beneficial effects on survival following infant vaccination with live vaccines against measles and BCG and may reduce all-cause mortality risk by 20–50% for those up to 5 years of age (76–79). It has been suggested that these effects may particularly benefit low-birth weight infants during the neonatal period because of reduced risk of respiratory infections and sepsis (76). A possible mechanism to explain these heterologous effects is the phenomenon of innate immune response training, which involves epigenetic reprogramming of monocytes leading to increased cytokine production in response to antigens unrelated to the original stimulus (80). Vaccination during pregnancy could therefore also have non-specific effects in the mother, fetus or newborn, and while this has not yet been formally investigated, to the best of our knowledge, one recent study did show that MF59-adjuvanted influenza vaccination during pregnancy led to an altered cytokine production profile in the nasal mucosa of 4-week-old infants compared with infants born to unvaccinated mothers (81). The underlying mechanisms, clinical implications, and the possible role of *in utero* priming remain to be determined.

Finally, it is also worth considering how the design and delivery of vaccination might affect the transplacental transfer of vaccine antigen, and the subsequent effect on fetal immune responses. First, the timing of exposure with respect to gestational age may have an impact on priming. Most studies to date have been conducted within the context of allergen exposure and have suggested that a cutoff at around 20–22 weeks of gestation might favor *in utero* sensitization to allergens (82, 83). Jones et al., for example, obtained blood samples from fetuses and premature babies to determine at what stage the fetal immune system produced a significant proliferative response to common allergens, including house dust mite, cat fur, and birth tree pollen (82). The authors found significantly higher PBMC proliferative response ratios in those infants who had been exposed to allergens beyond 22 weeks of gestation. A study by Vanderbeeken et al. focusing on *in utero* sensitization to tetanus vaccination also showed similar results, with detection of tetanus-specific IgM in cord blood occurring most often when women had undergone vaccination in the second or third trimester (56). Improving our understanding in this area, and establishing whether/when during gestation an optimum “window of opportunity” occurs for prenatal T cell priming may therefore inform the debate regarding the optimum time period for maternal vaccination. A number of other variables relating to vaccine design are also known to have an influence on vaccine efficacy and the resulting pattern of initial T cells responses, including vaccine type, dose, and route (4, 84). Vaccine adjuvants may also be used to guide the magnitude and type of adaptive response to vaccines (85). These factors may therefore conceivably have an effect on the quantity and/or quality of vaccine antigen that is able to transverse the placental barrier; however, direct research in this area is currently lacking.

## What Further Methods Could Be Employed to Study Vaccine Antigen Priming?

There is clearly a paucity of research on the topic of vaccine antigen priming, and the studies described above have been limited to using measurements of cord blood IgM and MHC tetramers. MHC tetramers offer a direct means of detecting antigen-specific T cells (59); however, the downside to their use is that their design requires considerable prior knowledge of the major pathogen epitopes recognized by human T cells, as well as the HLA type of each subject being studied (59, 60). This information might not be readily available for many vaccine studies and may limit their use in human trials. Another limitation of these studies is that they were conducted over short periods, and future research may benefit from repeated measurements of antigen-specific T cells over several months after birth, as this would provide further insight into the development of memory T cells.

Below we discuss other possible options for identifying vaccine-induced priming in future studies (including proliferation assays, cytokine-based assays, and animal studies) which could be undertaken without knowledge of specific antigen epitopes and MHC restriction elements. It should be noted that the interpretation of proliferative immune responses as supposed evidence of *in utero* priming still remains a subject of debate (43, 44). Furthermore, these measurements are indirect and may be prone to experimental variability due to differences in initial cell count and media/culture conditions (86–88).

### Proliferation Assays

One method that may be used to measure antigen-specific T cell proliferation is flow cytometry, of which assays include fluorescent dye dilution (using Oregon Green or carboxyfluorescein diacetate succinimidyl ester) (89, 90) and those detecting 5-bromo-2′-deoxyuridine (BrdU) using fluorochrome-conjugated antibody staining (91). Limitations of dye dilution include its cellular toxicity (92, 93) and sensitivity to pH and light (89) and the major limitation of using BrDU is that it inhibits cell cycle progression, meaning that only cells progressing through S-phase in less than 24 h will be identified (94).

A newer method which could be used in future studies to more reliably quantify antigen-specific T cell proliferation *in vitro* following vaccination is intracellular expression of the nuclear protein, Ki67 (94). To date, this protein has been used most commonly as a marker of tumor cell proliferation in cancer biology (95). Ki67 helps regulate cell division and is active throughout the cell cycle, but is not present in quiescent cells and during DNA repair, making it an ideal marker for determining the growth of a specific cell population (96). Furthermore, the assay does not require washing or incubation prior to culture, and cells are not exposed to toxic compounds. Soares et al. found that Ki67 was expressed in CD4+ and CD8+ T cells that had undergone *in vitro* proliferation in human whole blood or peripheral blood mononuclear cell assays with antigens, and the results correlated strongly with those demonstrated by traditional flow cytometry (94). T cells cultured in the absence of antigen did not express Ki67, and the assay was able to detect vaccine-specific CD4+ T cell proliferation after infant vaccination with tetanus toxoid. These findings support work by others who have demonstrated that intracellular Ki67 expression can be used to directly measure specific effector T cell responses induced by vaccination *ex vivo*, or after *in vitro* cell culture (97–99). One limitation of Ki67 as a marker is, unlike using dye dilution assays, one cannot calculate the number of proliferation cycles that have occurred, and therefore cannot estimate the original number of precursor cells (100).

### Cytokine-Based Assays

Cytokine-based assays are another possible option for future studies to characterize the immunological response to vaccines in pregnancy, as naïve and memory T cells each display distinct cytokine signatures. Memory cells produce cytokines such as IFN-γ within 20 h following antigen challenge, whereas naïve T cells must first undergo proliferation and differentiation before they can express such cytokines (101, 102). Traditional cytokine-based assays include enzyme-linked immunosorbent assay, and cytometric bead array, and more recently, enzyme-linked immunosorbent spot (ELISpot), FluroSpot, and intracellular cytokine staining (ICS) assays have been developed which can measure cytokine production on a per-cell basis (88). These measurements can be performed on whole blood or cryopreserved PBMCs and allow one to quantify functional populations of antigen-specific memory T cells (103, 104). ELISpot in particular has emerged as one of the most reliable methods of evaluating human immune responses to vaccines (105, 106). When using frozen PBMCs from the same donors, however, tetramer assays have been shown to have better precision and linearity than ICS or ELISpot (107).

### Animal Studies

Finally, animal models may offer the opportunity for more in-depth *in vivo* studies to probe the mechanics of *in utero* sensitization. Various species have been used to study the safety and immunogenicity of maternal vaccination to date (108); however, to the best of our knowledge, this approach has not yet been used to formally investigate the concept of priming following vaccination. T cell priming following exposure to infectious disease has been demonstrated by work in rodents, including an elegant study by Rahman et al. (10). They demonstrated that mycobacterial antigens administered to mothers during the second week of gestation were transported across the placenta, and that their offspring displayed higher specific T cell responses compared with offspring of untreated mothers. Antigen tracing was accomplished using fluorescent nanocrystals, and antigens conjugated with fluorescent Qdot were visible on the placental tissue as well as on the fetal tissue. Animal models involving real-time *in vivo* antigen tracing therefore offer an exciting avenue for future research. One downside of murine models is that B cell priming cannot be studied, as the appearance of B cells in mice and rats is delayed compared with humans and no B cells have been observed before birth.

## Conclusion

Increasing evidence suggests that exposure to maternal infection *in utero* may “prime” the developing immune system, even in the absence of infant infection, and some evidence suggests that this may also occur following vaccination. While this exciting field of research continues to expand, our understanding of the underlying mechanisms remains poor, and further work is required to elucidate the possible clinical implications. It is possible that *in utero* priming following vaccination could benefit the neonate by providing protection independent of antibody-mediated passive immunity; however, the possible effects of vaccination on subsequent infant vaccinations, their potential “non-specific” effects, and how the design and timing of vaccination may affect prenatal priming remain important questions to answer.

Looking forward, researchers should consider other possible options for quantifying antigen-specific T cells to establish firm evidence of priming following vaccination, as controversy remains over whether observed cell responses necessarily reflect *in utero* sensitization. We have discussed the use of MHC tetramers, as well as other novel proliferation and cytokine-based assays, and animal models using *in vivo* antigen tracing. Future research platforms would benefit from multidisciplinary collaborations and utilizing various placental models. Improving our understanding of the perinatal and neonatal immune systems is crucial for improving infant survival rates and the optimization of vaccination in pregnancy and in early life, especially in developing countries where the burden of infectious disease is the highest.

## Author Contributions

CW designed and wrote the article. CJ conceived, designed, and critically revised the article.

## Conflict of Interest Statement

CW and CJ are investigators for clinical trials done on behalf of University of Southampton and University Hospital Southampton NHS Foundation Trust, sponsored by various vaccine manufacturers including Novavax for a respiratory syncytial virus vaccine, but receive no personal funding for these activities.
